# The Italian landscape of robotic-assisted kidney transplantation: results from a national survey

**DOI:** 10.1007/s13304-026-02561-6

**Published:** 2026-06-01

**Authors:** C. Guidetti, S. Serni, U. Boggi, A. Solinas, F. Tanese, A. Lauterio, A. Volpe, B. Catellani, G. P. Guerrini, T. M. Manzia, D. Lorenzin, A. Puzziello, M. Romano, M. Spada, A. Antonelli, G. Carcano, N. Bossini, M. Iaria, M. Vivarelli, M. Rossi, P. Rigotti, L. Furian, R. I. Troisi, S. Di Sandro, F. Di Benedetto

**Affiliations:** 1 Hepato-Pancreato-Biliary Surgery and Liver Transplantation Unit, University Hospital of Modena “Policlinico”, University of Modena and Reggio Emilia, 41124 Modena, Italy; 2 Department of Experimental and Clinical Medicine, University of Florence, Florence, Italy; 3 Division of General and Transplant Surgery, University of Pisa, Pisa, Italy; 4 Department of Urology, Kidney Transplant and Robotic Surgery, ARNAS G. Brotzu, Cagliari, Italy; 5 Urology, Andrology and Kidney Transplantation Unit, Department of Regenerative and Precision Medicine, University of Bari “Aldo Moro”, Bari, Italy; 6 General Surgery and Abdominal Organ Transplantation, ASST Grande Ospedale Metropolitano Milano Niguarda, Milan, Italy; 7 Division of Urology, Department of Translational Medicine, Maggiore Della Carità Hospital, University of Eastern Piedmont, Novara, Italy; 8 Clinical and Experimental Medicine PhD Program, University of Modena and Reggio Emilia, Modena, Italy; 9 Hepatobiliary Surgery and Transplant Unit, Department of Surgical Sciences, University of Rome Tor Vergata, Rome, Italy; 10 Liver-Kidney Transplant Unit, Department of Medicine, University of Udine, Udine, Italy; 11 Dipartimento di Medicina, Chirurgia e Odontoiatria, Campus Universitario di Baronissi (SA) - Università di Salerno, AOU San Giovanni di Dio e Ruggi di Aragona, Salerno, Italy; 12 Department of Surgical, Oncological and Gastroenterological Science (DISCOG), Hepatobiliary and Pancreatic Surgery Unit - Treviso Hospital, University of Padua, Padua, Italy; 13 Division of Hepatobiliopancreatic Surgery, Liver and Kidney Transplantation, Ospedale Pediatrico Bambino Gesù IRCCS, Rome, Italy; 14 Unità Dipartimentale Trapianti di Rene, Dipartimento di Chirurgia ed Odontoiatria, Azienda Ospedaliera Universitaria Integrata di Verona, Verona, Italy; 15 Department of Medicine and Technological Innovation, University of Insubria, Varese, Italy; 16 Unit of Nephrology, ASST Spedali Civili di Brescia, Brescia, Italy; 17 Division of General Surgery, Transplant Surgery Unit, Department of General and Specialized Surgery, Parma University Hospital, Parma, Italy; 18 HPB Surgery and Transplantation Unit, Department of Clinical and Experimental Medicine, Polytechnic University of Marche, Ancona, Italy; 19 General Surgery and Organ Transplantation Unit, Sapienza University of Rome, Umberto I Polyclinic of Rome, Rome, Italy; 20 Pancreatic and Transplant Surgery, IRCCS San Raffaele Scientific Institute, Vita-Salute San Raffaele University, Milan, Italy; 21 Kidney and Pancreas Transplantation Unit, Department of Surgical, Oncological and Gastroenterological Sciences, University of Padova, Padua, Italy; 22 Division of Minimally Invasive and Robotic HPB Surgery and Transplantation Service, Federico II University Hospital, Naples, Italy

**Keywords:** Robotic assisted kidney transplant, Robotic surgery, Kidney transplant, National survey, Web-based survey

## Abstract

Robotic-assisted kidney transplantation (RAKT) is an emerging minimally invasive alternative to open surgery that offers potential benefits in selected recipients. Despite increasing international interest, its implementation across transplant centers remains variable. This study aims to describe the current status, challenges, and perspectives on RAKT in Italy through a nationwide survey. A web-based questionnaire was distributed to all active kidney transplant centers in Italy. The survey collected data on institutional experience, surgical techniques, intraoperative technologies, perceived barriers, and future directions. Responses were anonymized and analyzed descriptively. Twenty-one out of 39 centers responded (54%). Nine centers were actively performing RAKT at the time of the survey. The median number of RAKT cases per center was 5 (range 1–85), with Da Vinci Xi being the most used platform. Intraoperative tools such as indocyanine green fluorescence and robotic Doppler were variably adopted to verify graft perfusion. Reported operative times ranged from 143 to 410 min (median 215 min), with low conversion rates. Main barriers to adoption included limited access to the robotic platform and lack of structured training. All centers supported the creation of a national registry and expressed interest in collaborative studies. This survey provides the first national overview of RAKT in Italy, highlighting both technical feasibility and structural challenges. The findings support the need for coordinated training efforts, shared data, and policy-level initiatives to foster equitable access to robotic transplant surgery.

**Supplementary Information:**

The online version contains supplementary material available at https://doi.org/10.1007/s13304-026-02561-6.

## Introduction

Robotic-assisted kidney transplantation (RAKT) has emerged over the past decade as a minimally invasive alternative to conventional open kidney transplantation (OKT), offering potential advantages in terms of reduced postoperative pain, smaller incisions, fewer wound-related complications, and faster recovery, while maintaining comparable renal function and graft survival outcomes [1–3].

These benefits have been consistently reported in the literature. A recent meta-analysis of 11 comparative studies (482 RAKT vs. 1316 OKT) demonstrated that RAKT was associated with significantly lower rates of surgical site infections (risk ratio [RR] = 0.15), symptomatic lymphocele (RR = 0.20), postoperative pain (mean difference ≈ − 1.4 points), shorter incision length (mean difference ≈ − 8.5 cm), and reduced hospital stay (mean difference ≈ − 1.7 days), without compromising renal function or patient and graft survival [4].

Similar findings were confirmed by a more recent meta-analysis including 16 studies and 2555 patients, which highlighted reduced intraoperative blood loss, lower pain scores, and decreased SSI rates, particularly in obese recipients, with no significant differences in functional outcomes compared to OKT [5].

Despite these encouraging results, RAKT remains a technically demanding procedure characterized by a substantial learning curve. Evidence from high-volume European centers suggests that approximately 35 cases are required to achieve reproducibility in operative times, complication rates, and functional outcomes [6]. In this context, dedicated simulation-based training models have been developed to facilitate skill acquisition, optimize the learning curve, and support wider dissemination of the technique [7].

At the European level, several multicenter prospective observational studies, including those conducted by the ERUS RAKT working group, have reported favorable perioperative outcomes and acceptable mid-term functional results across multiple institutions, confirming the feasibility and safety of the robotic approach in experienced hands [8].

One of the main technical concerns traditionally associated with RAKT has been the potential for prolonged warm ischemia time; however, this issue has been effectively addressed by some centers through the adoption of regional hypothermia techniques, such as the intra-abdominal application of granular ice [9].

Although the literature also reports favorable outcomes in technically challenging scenarios, including grafts with multiple renal vessels, the diffusion of RAKT remains fragmented and largely confined to selected high-volume centers [10]. Moreover, robust evidence from randomized controlled trials comparing RAKT and OKT is still lacking, although at least one randomized study protocol aimed at evaluating surgical complication reduction has recently been registered [11].

Despite the growing international experience with RAKT, the organizational and structural context in which this technique is implemented varies substantially across countries. Italy represents a particularly informative setting, characterized by a heterogeneous distribution of kidney transplant centers, variable access to robotic surgical platforms, and non-uniform availability of structured training pathways. These factors may significantly influence the adoption, diffusion, and sustainability of RAKT programs, making a national-level assessment especially relevant from both organizational and health policy perspectives.

To address this gap, we conducted a nationwide survey involving all kidney transplant centers in Italy, with the aim of evaluating the current extent of RAKT adoption, surgical protocols, indications and contraindications, logistical challenges, and interest in future multicenter coordination. This study reports the results of the first structured national survey on robotic-assisted kidney transplantation in Italy.

## Materials and methods

### Study design and objectives

This study was designed as a national cross-sectional survey targeting all kidney transplant centers in Italy. The primary objective was to assess the current adoption of RAKT, identify technical and logistical models, evaluate institutional barriers, and explore future directions including training, research, and the creation of a dedicated national registry.

### Survey development

The questionnaire was developed by a panel of transplant surgeons with experience in robotic surgery and kidney transplantation. It consisted of 4 sections (33 questions total), including both multiple-choice and open-ended formats.

These are the 4 sections:

1. Background
2. Surgical Experience with RAKT and Technical Preferences
3. Perceived Barriers, Training, and Indications
4. Logistical Aspects and Interest in Collaboration

The survey explored topics such as indications for RAKT, platform availability, techniques used for vascular anastomosis, the role of ICG and intraoperative Doppler ultrasound, management of multiple vessels, and the need for proctoring or structured training programs.

The questionnaire was pilot-tested among three transplant surgeons with experience in robotic surgery and kidney transplantation to assess clarity, relevance, and completeness. Feedback obtained during this pilot phase was used to refine question wording and structure prior to national dissemination.

The present survey was conceived as a baseline assessment intended to inform future longitudinal data collection, particularly through the development of a national RAKT registry.

The complete questionnaire is available as Supplementary Material.

### Distribution and data collection

The survey was distributed via email using a secure link to a Google Forms platform. No personal, patient-related, or institution-identifiable data were collected through the survey, and responses were recorded anonymously.

Invitations were sent to transplant program directors or robotic surgery leads at each of the 41 Italian centers authorized to perform kidney transplantation, as identified through the official CNT (Centro Nazionale Trapianti) database and updated contact lists.

Data collection was open from 16 April 2025 to 15 May 2025. Given the voluntary nature of survey participation, a degree of response bias cannot be excluded. To mitigate this risk, responses were collected anonymously and two reminder emails were sent to non-responding centers prior to the final deadline.

### Data analysis

Responses were collected anonymously and analyzed descriptively. Given the exploratory aim of the study, the limited number of responding centers, and the heterogeneity in institutional characteristics, the analysis was intentionally restricted to descriptive statistics in order to avoid overinterpretation of the data.Categorical variables were reported as frequencies and percentages. No inferential statistics were performed, given the exploratory and descriptive nature of the study.

The study did not involve patients or clinical data and was therefore exempt from ethical committee approval.

## Results

The results presented below represent a descriptive, self-reported snapshot of current practices and are intended to provide a national baseline for future multicenter studies and policy planning rather than to support inferential analyses.

### Center participation and general adoption

A total of 21 transplant centers out of 39 invited (54% response rate) participated in the national survey (Table 1). Among these, 9 centers (43%) reported having performed at least one RAKT.

**Table 1:** Characteristics of participating centers and RAKT adoption

Characteristic	Centers performing RAKT (n = 9)	Centers not performing RAKT (n = 12)
Centers with robotic platform	9	5
Centers performing RAKT	9	0
Median number of RAKT procedures (range)	6 (1–85)	N/A
Use of living donor transplantation	8/9 (89%)	10/12 (83%)
Use of deceased donor transplantation (for RAKT centers data refers to use in the setting of RAKT)	DBD: 3/9 (33%); DCD: 1/9 (11%)	DBD 12/12 (100%); DCD 12/12 (100%)
Need for dedicated RAKT training	9/9 (100%)	11/12 (92%)
Interest in national RAKT registry	9/9 (100%)	12/12 (100%)

Although the overall response rate among Italian kidney transplant centers was approximately 50%, all centers known to have performed robotic-assisted kidney transplantation at the time of the survey responded, ensuring complete coverage of active national RAKT experience.

The earliest reported RAKT procedure was performed in 2010 at the University of Pisa and has been previously described as the first European case of robotic kidney transplantation [12].

Subsequent adoption occurred gradually, with additional centers initiating RAKT programs from 2013 onward and a clear acceleration observed between 2021 and 2024. Most centers introduced RAKT within 0–3 years after implementing robotic surgery, with a mean interval of 1.5 years between the two milestones; this finding reflects only a temporal association without any cause-effect relation.

Among the 9 centers that provided a quantitative estimate, the median number of RAKT procedures performed was 5, with a wide inter-center variability (range: 1 to 85 procedures). While some centers are still in an early phase of adoption, others have reached substantial case volumes, indicating growing institutional experience and consolidation of the technique.

Reported RAKT case numbers reflect the cumulative experience of each center up to the time of survey completion, with procedures performed over variable time spans depending on center experience and program maturity.

### Surgical technique and logistics

Among the 9 centers currently performing RAKT, the Da Vinci Xi platform was the most widely used (n = 8), followed by Da Vinci Si, which was employed in the earliest European case and has since been upgraded to Xi. All centers reported using a supine position, with the majority favoring a Trendelenburg tilt (n = 7), and two centers adopting alternative setups such as flat supine or flank elevation.

Techniques for intraoperative verification of optimal graft perfusion were variably adopted across centers. Indocyanine green (ICG) fluorescence was used routinely in 3 centers and selectively in 3 others, while 3 centers did not use it. Robotic Doppler ultrasound was more widely implemented, with 6 centers reporting routine use, 2 using it selectively, and only 1 center not employing it at all.

Regarding vascular anatomy, all centers performing RAKT reported that multiple renal vessels are not considered an absolute contraindication, but rather are evaluated on a case-by-case basis according to anatomical complexity and team experience.

The reported skin-to-skin operative time varied considerably across centers, ranging from 143 to 410 min, with a median duration of 215 min.

Finally, 6 out of 9 centers (67%) reported conducting a dedicated clinical and imaging follow-up protocol for RAKT recipients, while the others followed standard post-transplant care.

Importantly, 16 out of 21 total responding centers (76%)—including both those performing and not yet performing RAKT—identified access to the robotic platform and intra-hospital logistics as a relevant barrier to the implementation or expansion of RAKT programs.

As part of the learning curve analysis, centers were asked whether they performed donor nephrectomy, as this may contribute to robotic proficiency in RAKT. Robotic-assisted procurement was the predominant technique (n = 6), followed by laparoscopic procurement (n = 2), and both approaches being used depending on the case in one center.

### Clinical outcomes

Reported intraoperative complication rates were generally low across centers. While 2 centers explicitly reported no complications, others cited isolated events such as a venous laceration or technical challenges related to graft placement, particularly in early cases. No center reported a standardized intraoperative complication rate exceeding 5%.

As for conversion to open surgery, 7 out of 9 centers (78%) reported a 0% conversion rate. One center reported a rate below 5%, and another above 10%.

Postoperative vascular and urological complications were similarly rare. The majority of centers (n = 6) reported no such complications to date.

Postoperative hospital stay was reported in categorical ranges. Most centers (5 out of 9) indicated a mean length of stay between 8 and 10 days, while the remaining reported either 5–7 days (n = 3) or less than 5 days (n = 1). Direct comparison with national open kidney transplantation length-of-stay data was not performed, as such data were not collected within the scope of the survey.

### Barriers and perspectives

Several institutional and organizational barriers to the implementation or expansion of RAKT programs were identified by the responding centers. The most frequently cited obstacles included limited access to the robotic platform, intra-hospital logistical constraints, lack of structured training programs, and skepticism from other surgical teams or hospital management. The type of perceived barriers did not substantially differ between centers actively performing RAKT and those not yet performing the procedure; therefore, barriers are reported jointly rather than stratified by center activity status.

Regarding initial implementation, 5 centers reported having performed their first RAKT with the in-theatre presence of a proctor, while 4 centers proceeded independently.

When asked about training needs, 20 out of 21 centers (95%) expressed a clear need for dedicated RAKT-specific training, while only one center reported no such requirement.

The vast majority of centers expressed strong interest in national coordination efforts. Specifically, 18 centers declared interest in joining a national multicenter study, one center was undecided, and only one declined participation.

Furthermore, all 21 centers (100%) supported the creation of a national RAKT registry and expressed willingness to receive updates and participate in future collaborative initiatives.

## Discussion

The survey findings have been interpreted in terms of organizational models, training needs, and policy implications relevant to the diffusion of RAKT at a national level.

The Italian national survey on robotic-assisted kidney transplantation (RAKT) revealed a still fragmented adoption of the technique, despite increasing international evidence supporting its safety and efficacy. In particular, centers that have already adopted RAKT reported promising outcomes, with very low conversion rates and manageable complication profiles.

Several recipient populations could particularly benefit from the minimally invasive nature of RAKT. Among these, obese patients—traditionally at higher surgical risk—have shown substantial advantages [5].

Similarly, data from Kishore et al. [1] support the safe use of RAKT in obese recipients, showing comparable long-term outcomes to open transplantation.

These findings are consistent with the international literature, where RAKT has demonstrated high feasibility, low perioperative morbidity, and excellent functional outcomes. The self-reported data from Italian centers participating in this survey appear to align with these published results, further supporting the broader applicability of the technique, but require formal evaluation, especially regarding long term functional outcomes.

From a health policy perspective, access to robotic platforms remains one of the major structural obstacles. Kidney transplantation itself plays a central role in reducing long-term healthcare expenditures associated with end-stage kidney disease [13].

Although robotic surgery entails substantial upfront costs, available evidence suggests that postoperative length of stay may be comparable between robotic-assisted and open kidney transplantation; therefore, the overall economic impact of RAKT remains uncertain and highly context-dependent, in the absence of formal cost-effectiveness analyses [14].

Training and institutional readiness are also key factors. The survey highlighted the need for structured education: 95% of centers expressed the need for specific RAKT training, and many started their programs under proctoring. Simulation-based training models, proctoring, and stepwise implementation strategies may represent effective tools to support safe, reproducible, and scalable adoption of RAKT across centers. An interesting model have been recently published by the European Association of Urology [15].

The experience gained from robotic donor nephrectomy—already performed in several centers—may serve as a technical bridge toward RAKT implementation.

The unanimous interest in establishing a national registry and the widespread support for a multicenter collaborative study confirm a strong willingness to coordinate efforts. These initiatives would allow for:

- Shared guidelines
- Standardized training
- Real-world cost-effectiveness evaluations, and
- Broader access to innovation for fragile patient groups

Some limitations are inherent to the design of the study, including its cross-sectional nature, self-reported data, and lack of centralized clinical outcome validation. The absence of inferential or comparative analyses, such as comparisons between centers performing and not performing RAKT, represents a further limitation of the study and reflects its exploratory design. Moreover, the low response rate and the limited number of centers performing RAKT may reduce the generalizability of the findings.

In this perspective, the present survey may be regarded as a first exploratory phase, providing the organizational foundation for subsequent national registry-based studies integrating survey data with prospective clinical outcomes.

## Supplementary Information

Below is the link to the electronic supplementary material.

## Data Availability

The datasets generated and analyzed during the current study are available from the corresponding author upon reasonable request. An anonymized version of the survey responses can be shared for academic and non-commercial purposes, in compliance with privacy regulations.
