# Supplementary material for: The Italian landscape of robotic-assisted kidney transplantation: results from a national survey
Source: Updates Surg. 2026 Jun 1;78(5):1929–34. doi: 10.1007/s13304-026-02561-6 (PMC13538201; doi:10.1007/s13304-026-02561-6)
Supplement: Supplementary file 1 [file 13304_2026_2561_MOESM1_ESM.docx]

# Supplementary Material

## National Survey on Robotic-Assisted Kidney Transplantation (RAKT) in Italy

National survey addressed to Italian kidney transplant centers to evaluate the diffusion, surgical technique, results, and future perspectives of robotic-assisted kidney transplantation.

**SECTION 1 – Diffusion of the technique and surgical background**

1. Has your center ever performed robotic-assisted kidney transplantation (RAKT)?

- Yes, currently active
- Yes, but currently suspended
- No, never performed
- Planned / under evaluation

2. In which year was the first RAKT performed in your center?

(Open answer)

3. In which settings is RAKT performed in your center?

- Living donor
- Deceased donor after brain death (DBD)
- Deceased donor after circulatory death (DCD)
- Not applicable

4. How many RAKT procedures has your center performed in total to date?

(Open answer)

5. Did your center have previous experience in robotic surgery before introducing RAKT?

- Yes, in urologic surgery
- Yes, in general surgery
- Yes, in both
- No
- Not applicable

6. Does your center perform living donor nephrectomy, and with which technique?

- Open
- Laparoscopic
- Robotic
- Other
- Not applicable

7. How much time elapsed between the introduction of robotic surgery and the first RAKT?

(Open answer)

8. After how many cases did you perceive a significant improvement in surgical technique?

- 1–3 cases
- 4–6 cases
- 7–10 cases
- >10 cases
- Not yet achieved
- Not applicable

9. Was the first RAKT performed with the support of a proctor?

- Yes, on-site
- Yes, remote
- No
- Not applicable

**SECTION 2 – Surgical technique**

10. Which robotic platform is used for RAKT in your center?

- Da Vinci Si
- Da Vinci X
- Da Vinci Xi
- Da Vinci SP
- Other

11. Who performs the RAKT procedure?

- Transplant surgeon
- Urologist
- General surgeon
- Multidisciplinary team

12. Is the operating team always the same?

- Yes
- Partially
- No

13. Usual patient positioning:

- Supine
- Supine with Trendelenburg
- Lateral decubitus
- Other

14. Graft insertion site:

- Pfannenstiel incision
- Peri-umbilical incision
- Paramedian incision

15. What access temporary occlusion system is used?
    - Alexis ™
    - GelPort™
    - GelPoint™
    - Other
16. Type of vascular anastomosis:

- Fully robotic
- Hand-Assisted

17. Type of ureterovesical anastomosis:

- Robotic
- Extracorporeal

18. Use of ICG fluorescence:

- Yes, routinely
- Yes, selected cases
- No

19. Use of intraoperative ultrasound

- Yes, routinely
- Yes, selected cases
- No

**SECTION 3 – Clinical and surgical outcomes**

20. Total number of RAKT procedures performed

(Open answer)

21. Mean operative time

(Open answer)

22. Which are the condition favoring the robotic approach over standard open procedure?
    - Recipient obesity
    - Complex recipient anatomy
    - Living donation
    - Logistics
    - Other
23. Are multiple renal vessels considered a contraindication?

- Yes
- No
- Case-dependent

24. Peculiar intraoperative complication

(Open answer)

25. Conversion rate to open surgery:

- 0%
- <5%
- 5–10%
- >10%

26. Mean postoperative length of stay:

- <5 days
- 5–7 days
- 8–10 days
- >10 days

27. Overall assessment compared to open technique:

- Better
- Comparable
- Worse
- Not yet evaluable

28. Do you run dedicated follow-up in RAKT patients
    - Systematically
    - Selectively
    - Standard follow up

**SECTION 4 – Barriers and future collaborations**

29. Main perceived barriers (max 3):

- Cost
- Limited robot access
- Lack of training
- Technical complexity
- Longer operative time
- Lack of guidelines
- Organizational issues

30. Training modalities considered most useful:

- Proctoring
- Simulation
- Observership
- Structured courses

31. Professionals reluctant to technique adoption
    - Transplant surgeons
    - Urologists
    - Anesthesiologists
    - Nephrologists
    - Administration
    - No resistance to introduction of RAKT
32. Interest in multicentric national study:

- Yes
- No

33. Are you in favor to the creation of national RAKT registry?
    - Yes
    - No
